# Uncovering newly identified aldehyde dehydrogenase 2 genetic variants that lead to acetaldehyde accumulation after an alcohol challenge

**DOI:** 10.1186/s12967-024-05507-x

**Published:** 2024-07-29

**Authors:** Freeborn Rwere, Joseph R. White, Rafaela C. R. Hell, Xuan Yu, Xiaocong Zeng, Leslie McNeil, Kevin N. Zhou, Martin S. Angst, Che-Hong Chen, Daria Mochly-Rosen, Eric R. Gross

**Affiliations:** 1 Department of Anesthesiology, Perioperative and Pain Medicine, School of Medicine, Stanford University, Stanford, CA 94305 USA; 2 Department of Chemical and Systems Biology, School of Medicine, Stanford University, Stanford, CA 94305 USA; 3 Present address: Department of Anesthesiology, University of California Davis, Davis, CA USA; 4 Present address: Department of Cardiology, The First Affiliated Hospital of Guangxi Medical University, Guangxi, China

**Keywords:** Genetic variant, Acetaldehyde, Alcohol, Aldehyde dehydrogenase 2 (ALDH2), Alcohol challenge, Enzyme kinetics

## Abstract

**Background:**

Aldehyde dehydrogenase 2 (ALDH2) is critical for alcohol metabolism by converting acetaldehyde to acetic acid. In East Asian descendants, an inactive genetic variant in ALDH2, *rs671*, triggers an alcohol flushing response due to acetaldehyde accumulation. As alcohol flushing is not exclusive to those of East Asian descent, we questioned whether additional ALDH2 genetic variants can drive facial flushing and inefficient acetaldehyde metabolism using human testing and biochemical assays.

**Methods:**

After IRB approval, human subjects were given an alcohol challenge (0.25 g/kg) while quantifying acetaldehyde levels and the physiological response (heart rate and skin temperature) to alcohol. Further, by employing biochemical techniques including human purified ALDH2 proteins and transiently transfected NIH 3T3 cells, we characterized two newly identified ALDH2 variants for ALDH2 enzymatic activity, ALDH2 dimer/tetramer formation, and reactive oxygen species production after alcohol treatment.

**Results:**

Humans heterozygous for *rs747096195* (R101G) or *rs190764869* (R114W) had facial flushing and a 2-fold increase in acetaldehyde levels, while *rs671* (E504K) had facial flushing and a 6-fold increase in acetaldehyde levels relative to wild type ALDH2 carriers. In vitro studies with recombinant R101G and R114W ALDH2 enzyme showed a reduced efficiency in acetaldehyde metabolism that is unique when compared to E504K or wild-type ALDH2. The effect is caused by a lack of functional dimer/tetramer formation for R101G and decreased V_max_ for both R101G and R114W. Transiently transfected NIH-3T3 cells with R101G and R114W also had a reduced enzymatic activity by ~ 50% relative to transfected wild-type ALDH2 and when subjected to alcohol, the R101G and R114W variants had a 2-3-fold increase in reactive oxygen species formation with respect to wild type ALDH2.

**Conclusions:**

We identified two additional ALDH2 variants in humans causing facial flushing and acetaldehyde accumulation after alcohol consumption. As alcohol use is associated with a several-fold higher risk for esophageal cancer for the E504K variant, the methodology developed here to characterize ALDH2 genetic variant response to alcohol can lead the way precision medicine strategies to further understand the interplay of alcohol consumption, ALDH2 genetics, and cancer.

**Supplementary Information:**

The online version contains supplementary material available at 10.1186/s12967-024-05507-x.

## Background

**Fig. 1:**
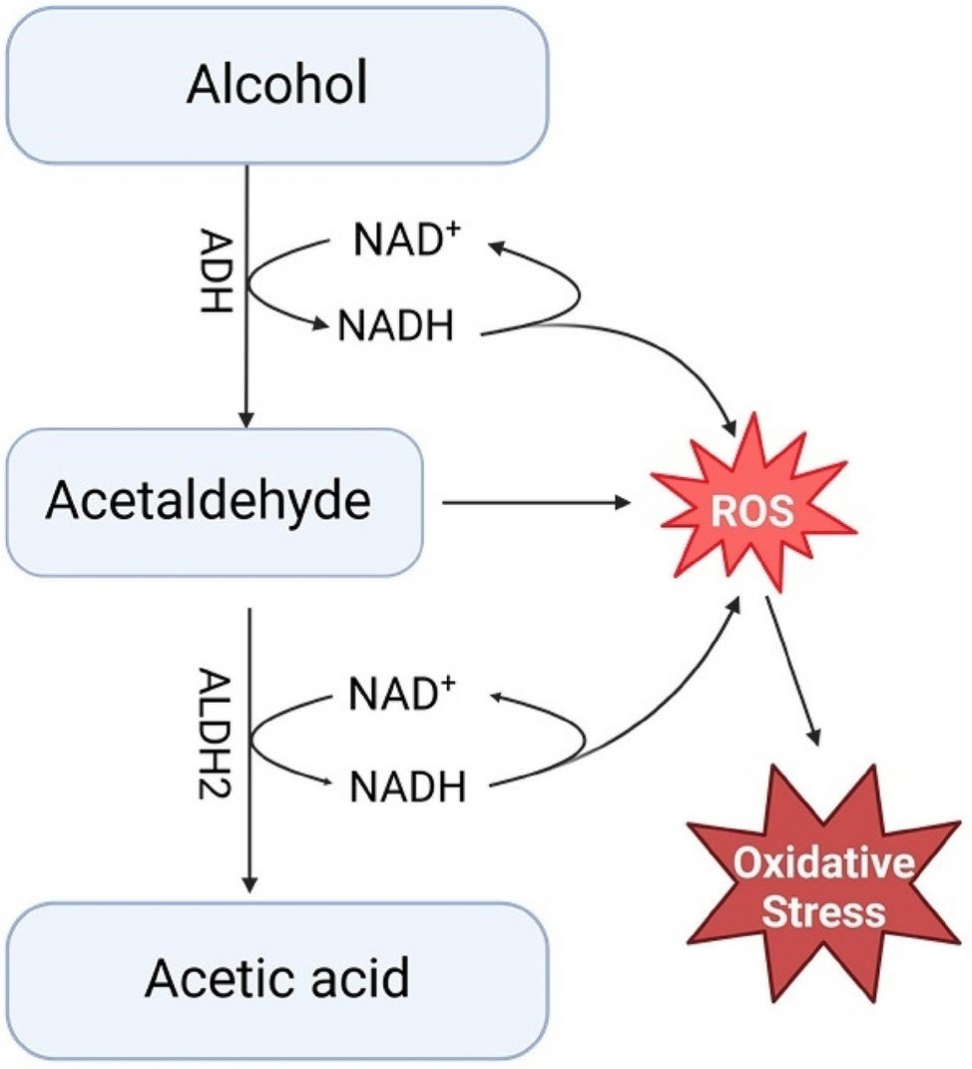
**Alcohol metabolism and reactive oxygen species generation.** Reactive oxygen species (ROS) are generated when ADH converts alcohol to acetaldehyde and when ALDH2 converts acetaldehyde to acetic acid. For both ADH and ALDH2, NADH is converted back to NAD^+^ and an electron is released reacting with oxygen to form ROS. Electrons from these steps can further react with dioxygen species (O_2_) to generate the superoxide (O_2_·^−^) and hydroxyl (OH.) radicals. Accumulation of acetaldehyde, which occurs with inactive ALDH2 variants, results in damage to the electron transport complexes leading to overproduction of ROS

Alcohol is consumed by at least 2 billion people world-wide annually [1, 2]. When alcohol is metabolized, acetaldehyde accumulation can lead to an overproduction of cellular reactive oxygen species (ROS); adding to the basal ROS production occurring when alcohol is metabolized to acetaldehyde and acetic acid [3, 4] (Fig. 1). Therefore, acetaldehyde accumulation is one of the important factors driving the pathophysiology of alcohol-induced cancer [5, 6]. Broadly, this biological process is not only important for alcohol-mediated carcinogenesis, but is also linked to the mechanism of drug-induced cellular toxicity [7–9].

In East Asian countries, ~ 40% of the population carry a genetic variant in the aldehyde dehydrogenase 2 (ALDH2) enzyme that metabolizes acetaldehyde to acetic acid [10, 11]. The ALDH2 genetic variant (ALDH2*2, *rs671*), causes acetaldehyde accumulation along with facial flushing and tachycardia after alcohol consumption [12]. The ALDH2*2 variant leads to an accumulation of acetaldehyde after alcohol consumption which contributes to an overproduction of ROS leading to carcinogenesis [13, 14]. Even moderate alcohol consumption for ALDH2*2 carriers increases the odds ratio for developing aerodigestive track cancer (2.61, 1.19–5.75) and esophageal cancer (3.12, 1.95–5.01) due to acetaldehyde accumulation from inefficient acetaldehyde metabolism [15, 16]. East Asia also has the highest world-wide incidence of esophageal cancer; likely due to the high frequency of the ALDH2*2 variant [17]. As nearly 540 million people of either sex carry the ALDH2*2 genetic variant, the frequency of ALDH2*2 carriers within the human population shines an initial light into understanding how alcohol use, an inactive ALDH2 genetic variant, and subsequent acetaldehyde-induced ROS production can lead to an increased risk for aerodigestive tract and esophageal cancers [12, 18–22].

Further, the genetic database gnomAD indicates there are ~ 570 missense ALDH2 variants occurring across all ethnicities which may cause a loss of function, gain of function, or no difference in ALDH2 enzyme activity. Whether other variants in ALDH2, besides *rs671* (E504K), cause facial flushing and acetaldehyde accumulation after alcohol consumption remain uncharacterized in humans. To accelerate this process of discovery, a non-invasive method to quantify acetaldehyde levels along with the physiological response to alcohol in humans is needed. Here, we developed in humans a non-invasive technique to test how ALDH2 variants alter acetaldehyde metabolism and the physiological response to alcohol. We then confirmed these findings using biochemical assays in vitro and in cell culture. This is important to understand considering the cancer risk associated with facial flushing occurring for the *rs671* variant [23, 24].

## Methods

For this manuscript, we performed a comprehensive literature review using PubMed, Google Scholar, and Science Direct covering years 1998–2024. The literature review served as the foundation for the manuscript including assisting with the formulation of the research question.

Prior to recruitment and testing of humans, IRB approval was obtained from Stanford University (IRB 46095). This study is a basic experimental study involving humans with the manipulation or task used (consuming alcohol) expressly used for measurement and is not an intervention. Written informed consent was received from participants prior to inclusion in the study. The experiments conformed to the principles set out in the WMA Declaration of Helsinki and the Department of Health and Human Services Belmont Report.

### Participant recruitment

Participants were recruited from flyers posted on the Stanford University campus in succession. The initial flyer recruited people who flush after they consume alcohol for an alcohol study. The flyer was then modified to recruit people only of non-Asian descent.

Participants were initially screened for exclusion using a questionnaire. Participants were also instructed to complete a CAGE questionnaire to screen out participants with potential alcoholism. Those participants eligible then provided a saliva sample for DNA extraction and purification (Quick-DNA Miniprep Kit, Zymo Research). DNA was amplified using 12 primers designed to cover the ALDH2 exome for sequencing (Supplemental Table 1). After amplification, samples were sent for Sanger sequencing (Eton Bioscience).

### Human alcohol challenge

Participants were selected for the alcohol challenge portion of the study based on genotyping results. At this stage, participants were also excluded if homozygous for *rs671* (secondary to the expected severity of the alcohol response). Regarding patient selection, the human volunteers for this study were healthy volunteers without a past medical history or taking any medications. None of the patients had a history of cardiovascular disease or diabetes mellitus. East Asian participants heterozygous for *rs671* (E504K) were called back for participation in the alcohol challenge. In addition, people without sequenced variants were also selected to closely match the age, sex and weight of the participants heterozygous for *rs671* (E504K). Additional participants were selected for the alcohol challenge study based upon genotyping results identifying people who were carriers of the ALDH2 variants including *rs747096195* (R101G) and *rs190764869* (R114W). Details regarding patient demographics are reported (Supplemental Table 2).

Participants were instructed prior to the alcohol challenge not to eat or drink for 2 h prior to testing. Testing was performed between 12pm and 4pm on a weekday. Upon arrival, patients were consented and weighed. To assess physiologic measurements, a five lead EKG was placed and EKG measurements were recorded (ADInstruments). Skin temperature was measured under the left cheek bone using liquid crystal technology (Crystaline II Temperature Indicators, Sharn Anesthesia). Baseline hemodynamic data including heart rate and skin temperature were collected. Prior to testing, a hand-held alcohol breath test meter confirmed no alcohol was consumed before testing (BACtrack Element Pro). Breath samples were collected by a 3 L Tedlar bag (Zefon International) that were sealed after collection. Breath samples were analyzed within 4 h of sample collection.

**Fig. 2:**
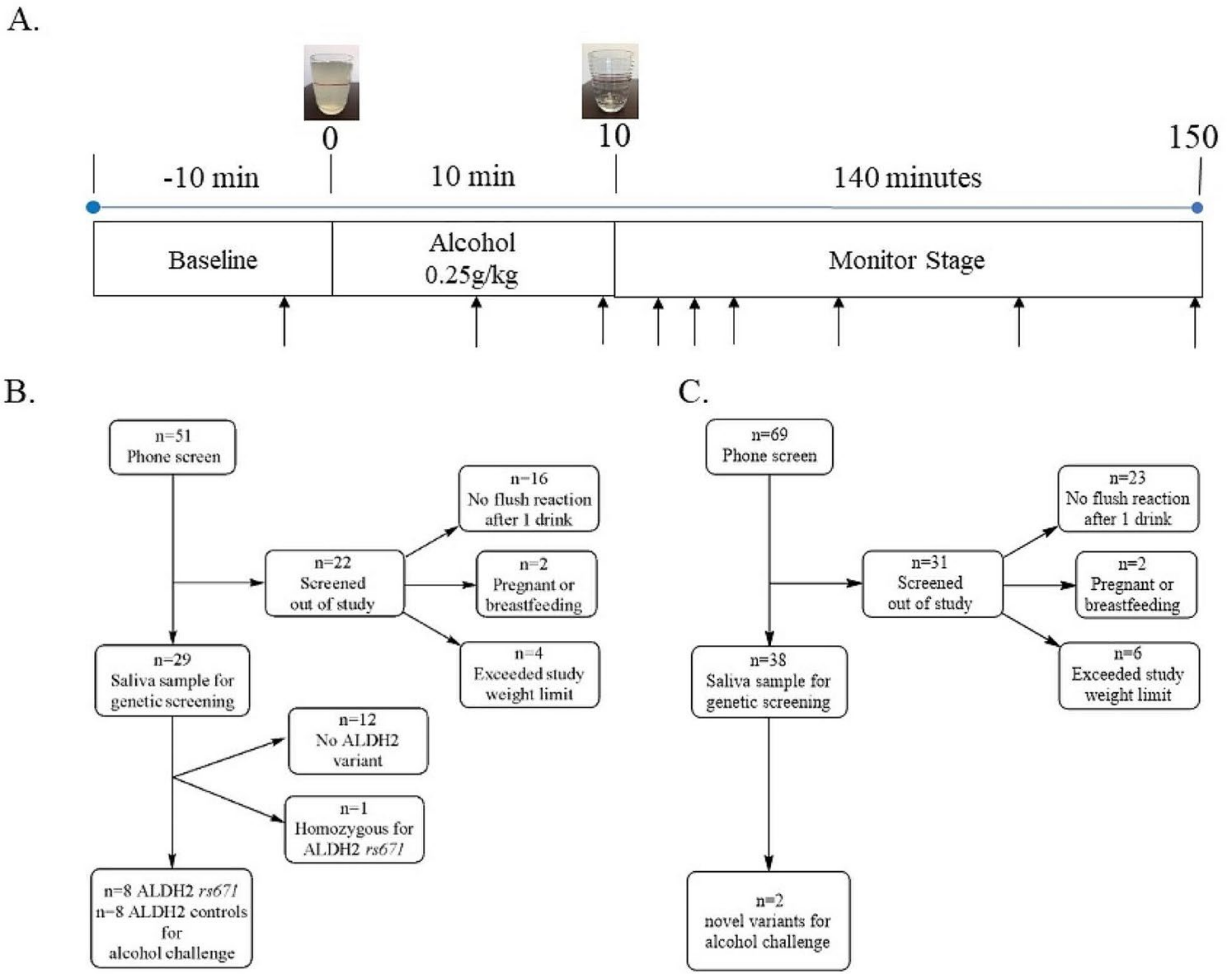
**Flowchart and experimental protocol for alcohol challenge. (A)** Alcohol challenge protocol. After 10 min baseline, subjects were given an alcohol challenge (0.25 g/kg); consumed over 10 min. Subjects were then monitored while measuring physiologic parameters (heart rate and skin temperature). **(B)** Recruitment of 16 subjects of East Asian descent for an alcohol challenge after genotyping. 8 subjects (4 male and 4 female) were selected for alcohol challenge with an ALDH2*1*2 genotype. Additionally, 8 subjects with an ALDH2*1/*1 genotype were age and sex matched to those with an ALDH2*1/*2 genotype. **(C)** Recruitment of subjects self-identified as non-East Asian descent. We identified 2 subjects that were carriers for uncharacterized genetic variants in ALDH2 that were subjected to an alcohol challenge

After establishing a baseline, participants were given a 415 mL (14oz) drink consisting of 0.25 g/kg Ketel One vodka, water, and Minute Maid lemonade. We chose vodka for the alcohol challenge to minimize the effect of congeners on our study [25]. The lemonade was used as a mixer. For reference in the United States, a standard drink is 14 g of alcohol which is equal to one 12 oz glass of 5% beer or one 5oz glass of 12% wine [26]. As an example, a 70 kg person in this study drank 17.5 g of alcohol. Participants consumed the first 7oz of the 14oz drink during the first 5 min of the study and the second 7oz during the second 5 min. After baseline measurements taken 3 min before alcohol consumption, physiologic measurements of heart rate, skin temperature, and breath metabolites were taken at 5, 10, 15, 30, 60, 90, 120, and 150 min during and after alcohol consumption (Fig. 2A). After completion of the study, a breath alcohol test and a standard field sobriety test were administered. The study lasted a total duration of 3 h. When collecting the data, we considered heart rate and facial skin temperature as the physiological measurements to analyze in relation to the acetaldehyde levels measured. Participants completing the alcohol challenge were given $125 compensation.

### Breath metabolite analysis

The breath metabolite acetaldehyde was measured from a Tedlar bag using selective ion flow mass spectrometry (Syft Voice Ultra, New Zealand). Soft ionization was carried out using H_3_O^+^ and NO^+^ with the mass and reaction ratios described for measuring acetaldehyde (Supplemental Table 3). Helium was used a carrier gas.

### *In Silico* ALDH2 Modeling

Protein data bank (PDB) entry 1O02 was imported into PyMOL (Schrödinger, Inc.) for molecular visualization of wild-type ALDH2 and to mutate R101 and R114 by using the protein mutagenesis function.

### ALDH2 site-directed variant construction

Protein Expression and Purification: Based upon genotyping results, site-directed mutagenesis of human ALDH2 was performed by PCR using a QuikChange site-directed mutagenesis kit (Agilent Technologies) and a kanamycin resistance template plasmid, pET-28a-c (+) containing full length cDNA of wild type ALDH2 gene. Oligonucleotides were used to generate ALDH2 variants (R101G, R114W and E504K) (Supplementary Table 4). The mutated plasmids were sequenced at Sequetech Corporation (Mountain View, CA) to check for the presence of the desired mutations and absence of unwanted base changes within the plasmids. Co-expression of wild-type ALDH2 and its site directed variants (R101G, R114W and E504K) was achieved using the pEDuet-1 vector plasmid (Millipore Sigma, Milwaukee, WI). Briefly, we cloned one copy of wild-type ALDH2 into the His-tag cloning site of the pETDuet-1 vector plasmid by EcoRI and HindIII and another copy of wild-type ALDH2 into the S-tag using NdeI and XhoI. For co-expression of wild-type ALDH2 with the variants, we cloned one copy of wild type ALDH2 into the His-tag cloning site as described above and the ALDH2 variants into the S-tag cloning site of pETDuet-1 plasmid using NdeI and XhoI. Co-expression of the recombinant proteins was carried out at 30 °C in the presence of GroEL chaperone using *E. coli* BL21 (DE3) host cells. Cells were induced with 0.5 M IPTG and grown for 16 h at 30 °C. Cells were recovered by centrifugation at 5000 rpm and recombinant proteins were lysed with B-PER complete bacterial protein extraction reagent (Thermo fisher Scientific, South San Francisco, CA). Protein purification was achieved with a His-trap nickel affinity column (Millipore Sigma, Milwaukee, WI). Following purification, the amount of protein was determined using the Pierce BCA Protein Assay Kit (Thermo fisher Scientific, South San Francisco, CA).

### Expression of wild-type ALDH2 and its variant enzymes in NIH-3T3 cells

Transient transfection of wild type ALDH2 and ALDH2 missense variants was performed in the NIH-3T3 cell line (BPS Bioscience #79,469) cultured in Growth 5 A medium. Once the 3T3 cells had reached 80% confluence, they were transfected with either the mammalian expression pCMV3-ALDH2-C-FLAG plasmid harboring wild-type ALDH2 or the ALDH2 variants. Additional cells were transfected with an empty pCMV3-C-FLAG vector. Transfection complexes were prepared using a 1:5 ratio (1 µg of plasmid/5µL of Lipofectamine 2000) in Opti-MEM-I-Reduced Serum Medium and incubated for 2 h. After incubation, the reduced serum medium was replaced with Growth 5 A medium and transfection was allowed to proceed for 72 h. Protein expression analysis was conducted 72 h post-transfection using an enzymatic activity assay as described below.

### Aldh2 enzymatic activity assay

NAD^+^ conversion to NADH in the presence of acetaldehyde was used to determine ALDH2 enzyme activity of purified recombinant protein (5 µg/mL) or total lysate (200 µg/mL) for cultured cells at pH = 9.0 and pH = 9.5, respectively as previously described [23]. For Alda-1 studies, wild type or ALDH2 variants were incubated with Alda-1 (20µM final concentration) for 2 min and assayed at pH = 9.0 as described elsewhere [27].

### Reactive oxygen species (ROS) production

For cellular ROS production, NIH-3T3 cells were seeded to 70–80% confluence. The cells were then incubated with 2,7-dichlorofluorescein diacetate (DCFDA, 5 µM) in basal medium at 37 °C for 30 min in the dark. After 30 min, fluorescence was measured at 37 °C (485/535 nm for excitation/emission) to measure the basal level of ROS (Growth 5 A medium was used a vehicle). The cells were then treated with 50 mM ethanol and fluorescence was again measured. Fluorescence intensity was normalized to the basal level for each protein. The dose of 50mM ethanol was chosen for this study because it is used in prior experimental studies when using cell culture assays studying the effects of alcohol [23, 28, 29]. This level is considered a physiologic exposure to a cell as in humans, the circulating blood ethanol levels are within the 10–15 mM range after 0.25 g/kg-0.75 g/kg alcohol consumption [30].

### ALDH2 cross-linking and western blot analysis

ALDH2 cross-linking was carried out by mixing bis(sulfosuccinimidyl)suberate (62.5 mM final concentration) with purified wild-type ALDH2 or ALDH2 variants (500 µg/mL) followed by adding SDS-loading buffer (5 µL, 120 mM Tris-HCl, pH 6.8, 4% (w/v) SDS, 20% (v/v) glycerol, 5% (w/v) bromophenol blue, 1% (w/v) DTT) in a total volume of 20µL at 4 °C [31]. MagicMark XP Western Protein Standard (ThermoFisher- Cat #: LC5603) was used to detect the molecular weight markers on western blot. The crosslinking mixture was analyzed by an 8% SDS-page gel (100 volts, 2h30min) at 4 °C and transferred to a nitrocellulose membrane (20 V, 16 h) at 4 °C. The next day membranes were washed with TBST buffer (4 times) and blocked with blotting-grade blocker (Biorad, Cat# 1,706,404) for 1 h. After 1 h, the membranes were probed using antibodies to ALDH2 (goat polyclonal, 1:1000, Abcam, Fremont, CA) or S-tag (rabbit monoclonal, 1:1000, Cell Signaling Technology, Danvers, MA) for 2 h and then washed 4 times with TBST buffer. Following washing, the membranes were incubated with anti-goat (1:2000 concentration, Abcam, Cat# ab97110) or anti-rabbit (1:2000 concentration, Cell Signaling Technology, Cat# 70,745) secondary antibodies for 1 h. After 1 h, the membranes were again washed with TBST buffer and proteins were visualized by SuperSignal West Pico PLUS Chemiluminescent Substrate (Thermo Fisher Scientific, South San Francisco, CA). Images were acquired with Azure Biosystems C300 for analysis.

### Statistical analysis

All data is presented as mean ± SEM. The number needed to recruit for this study is based upon prior studies examining differences in acetaldehyde metabolism in the blood for carriers of the *r671* variant versus non-carriers [30, 32, 33]. Based upon the dose of alcohol (0.25 g/kg) producing a 5-fold difference in blood acetaldehyde levels [30], we predicted that at a minimum there was a need to recruit 4 people per group to identify statistical differences between people that are wild type ALDH2 relative to people that are heterozygous for *rs671* (E504K). For analysis of differences in metabolites or physiological data, a two-way ANOVA followed by Bonferroni correction for multiplicity was used in order to compare each group to the control group or to compare measurements performed within a group over time. When comparing heart rate or skin temperature changes with acetaldehyde levels, a Pearson correlation test with a 2-tailed p-value was used. A two-tailed Student’s t-test was performed to compare the treated and untreated recombinant ALDH2 using Alda-1 and in ROS generation experiments. In addition, a one-way ANOVA was performed to determine the statistical difference of recombinant ALDH2 proteins or the ALDH2 transient transfection studies. Statistical analysis was performed using GraphPad Prism 6. *p < 0.01 was considered statistically significant between groups compared at the same time-point or end-point and ^*p* < 0.01 was considered statistically significant within groups with respect to vehicle or baseline.

## Results

### Human subjects studies

After genotyping human subjects, we recruited relevant participants to partake in an alcohol challenge. Initially, we recruited positive and negative controls for this study by recruiting those of Asian descent with and without the *rs671* (E504K) genetic variant. For this portion of the study, 58 people contacted the laboratory regarding participation. Of those, 51 people participated in the initial phone screen with 29 people providing a saliva sample for genotyping, and 16 people were given an alcohol challenge (Fig. 2A); 8 were heterozygotes for *rs671* and 8 were not (Fig. 2B). We then modified the protocol to recruit participants only of non-Asian descent. We had 69 people who underwent a phone screen to identify self-reporting flushing after ethanol exposure, 38 people provided saliva samples, and based on the genotyping results, we identified people heterozygous for *rs747096195* (R101G) or *rs190764869* (R114W).

**Fig. 3:**
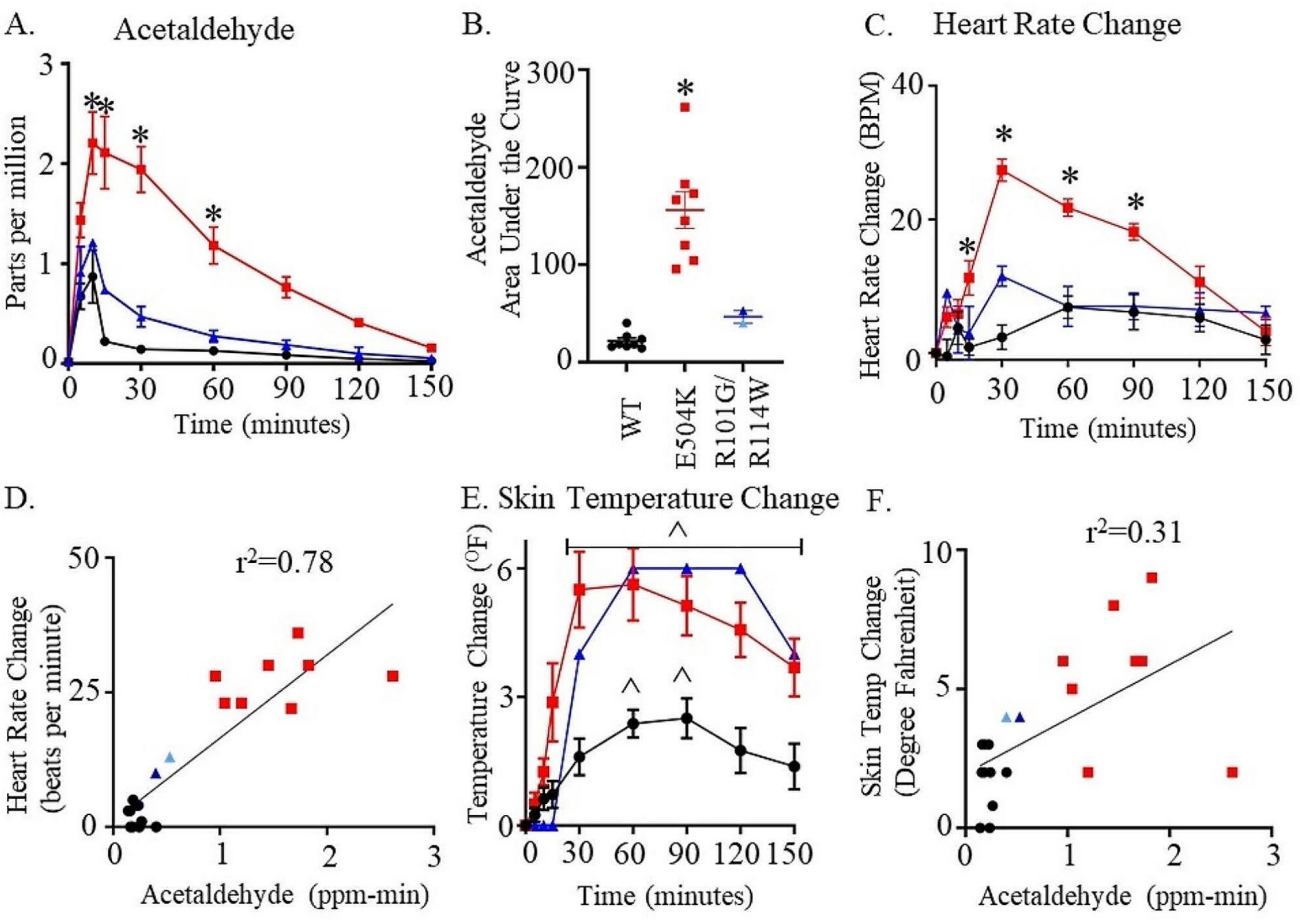
**Breath metabolite and physiologic characteristics after alcohol challenge. (A)** Breath concentrations (in parts per million) of acetaldehyde. **(B)** The total area under the acetaldehyde curve. **(C)** Heart rate change after alcohol consumption **(D)** Change in heart rate versus change in breath acetaldehyde concentration. **(E)** Skin temperature change after alcohol consumption and **(F)** Change in skin temperature versus change in breath acetaldehyde concentration. Black lines are wild type subjects, dark blue lines are R101G heterozygote, light blue lines are R114W heterozygote and red lines are E504K heterozygote subjects. *p < 0.01 by one-way or two-way ANOVA versus wild type subjects at the same time-points, ^*p* < 0.01 by two-way ANOVA with respect to baseline values within groups, *n* = 8/group for wild-type or E504K (4 male and 4 female) and *n* = 2/group (1 female R101G and 1 female R114W).

To explore the phenotype after alcohol consumption based on the ALDH2 genotyping from human subjects, we subjected participants who were heterozygous for *rs747096195* (R101G), *rs190764869* (R114W), and *rs671* (E504K) to an alcohol challenge and compared the response to those participants carrying wild type ALDH2. We considered the *rs747096195* and *rs190764869* variants together as these variants are within the same alpha helix for ALDH2 and each occur in a low frequency for humans. When comparing heterozygotes for *rs671* (E504K, ALDH2*1/*2) with respect to those with a wild type ALDH2 genotype, breath acetaldehyde levels peaked 9-fold 5 min after completing alcohol consumption (Figs. 3A and 15-minute time point measurement, peak: 2.1 ± 0.4^*^ versus 0.2 ± 0.3 ppm, *n* = 8/group). Acetaldehyde levels remained significantly elevated for 60 min after initial alcohol consumption (Fig. 3A, 1.2 ± 0.2* versus 0.13 ± 0.02 ppm, *n* = 8/group). Further, *rs747096195* (R101G) and *rs190764869* (R114W) accumulated acetaldehyde and took longer to metabolize acetaldehyde relative to those subjects that were wild type ALDH2 (Fig. 3A). This effect was not as prominent as compared to heterozygotes for *rs671* (E504K). The total area under the acetaldehyde curve was 6-fold greater for the ALDH2*1/*2 genotype versus the ALDH2*1/*1 genotype (Figs. 3B and 143 ± 14* versus 22 ± 3 ppm-minutes, *n* = 8/group). Further, total acetaldehyde had a two-fold accumulation for *rs747096195* and *rs190764869* (Figs. 3B and 46 ± 7 ppm-minutes).

Concomitantly, heart rate markedly increased in ALDH2*1/*2 participants relative to ALDH2*1/*1 participants peaking at 30 min (Figs. 3C and 104 ± 3* versus 73 ± 4 beats per minute, *n* = 8/group). No relative changes in heart rate occurred for those participants carrying *rs747096195* (R101G) or *rs190764869* (R114W) variants (Fig. 3C). The change in heart rate versus change in breath acetaldehyde concentration also correlated during peak acetaldehyde concentrations (Fig. 3D, r^2^ = 0.78). Additionally, skin temperature changes relative to baseline significantly increased for *rs671* at 30 min followed by *rs747096195* (R101G) and *rs190764869* (R114W) at 30 min after the start of alcohol consumption (Fig. 3E). However, skin temperature changes had less of a correlation with acetaldehyde levels measured at 30 min (Fig. 3F, r^2^ = 0.31).

### *In vitro* and cell culture studies

**Fig. 4:**
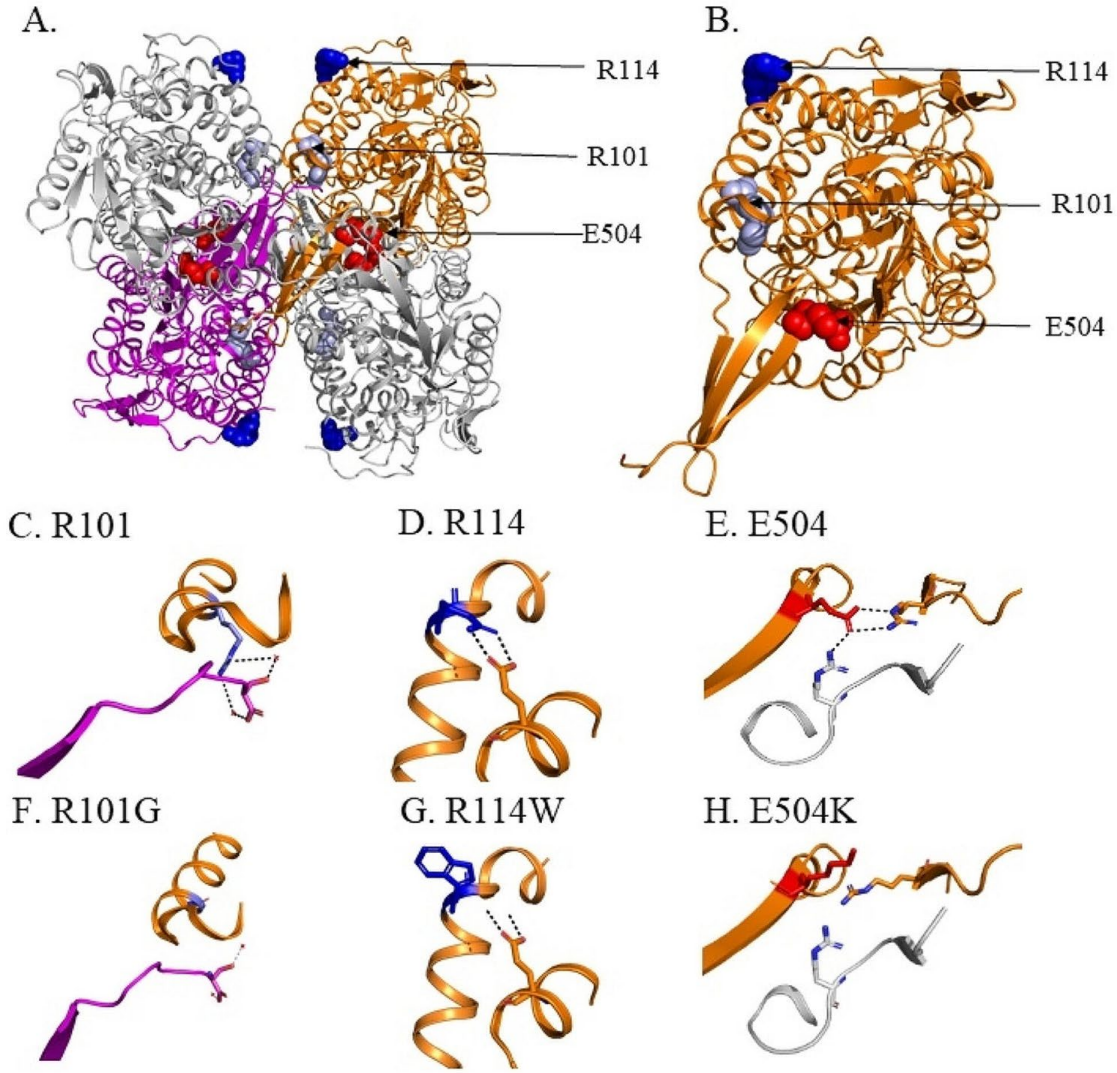
**Location of R101G R114W and E504K variants in ALDH2 and impact of these variants. (A)** Crystal structure of ALDH2, with 3 amino acids within the tetramer highlighted (R101 (light blue), R114 (dark blue) and E504 (red)). **(B)** Location of R101 (light blue), R114 (dark blue) and E504 (red) in the ALDH2 subunit. H-bond interactions between **(C)** R101 with S517 of another subunit *via* conserved water. **(D)** R114 with E227 of the same subunit. **(E)** E504K with R281 of one subunit and R492 of another ALDH2 subunit. **(F)** The R101G variant leads to loss of H-bonds with S517 of the opposite subunit. **(G)** R114W variant leads to loss of H-bonds with E227 within the same subunit and **(H)** E504K leads to loss of H-bonds with R281 within the same subunit and R492 of another subunit at the dimer interface

When examining the crystal structure of ALDH2, R101G and R114W are located in distinct regions of the α-B helix that are separate from E504K (Fig. 4A and B). A closer look at the ALDH2 tetramer suggests that R101 is important in the interaction between two opposite ALDH2 subunits of the tetramer, while R114 is important in the interaction of α-B and α-E helices within the same monomer (Fig. 4C and D, respectively). In addition, E504 located in the oligomerization domain is important for the formation of homodimers at the dimer interface (Fig. 4E). In wild-type ALDH2, R101 of one subunit forms a hydrogen bond with S517 of the opposite ALDH2 subunit via a conserved water molecule, while R114 forms an H-bond with E227 of the same subunit (Fig. 4C and D). The E504 residue form hydrogen bonds with R281 of the same subunit and R492 of the adjacent dimer partner [34]. When R101 is mutated to a glycine, a hydrogen bond *via* a conserved water molecule is lost between R101 and S517 (Fig. 4F). Upon R114W mutation, the two hydrogen bonds between R114 of α-B helix and E227 of the adjacent α-E helix within the same subunit are lost (Fig. 4G). The E504K variant disrupts the hydrogen bonding network between E504 and residues, R281 and R492, leading to a change in conformation of these residues at the dimer interface (Fig. 4H).

**Fig. 5:**
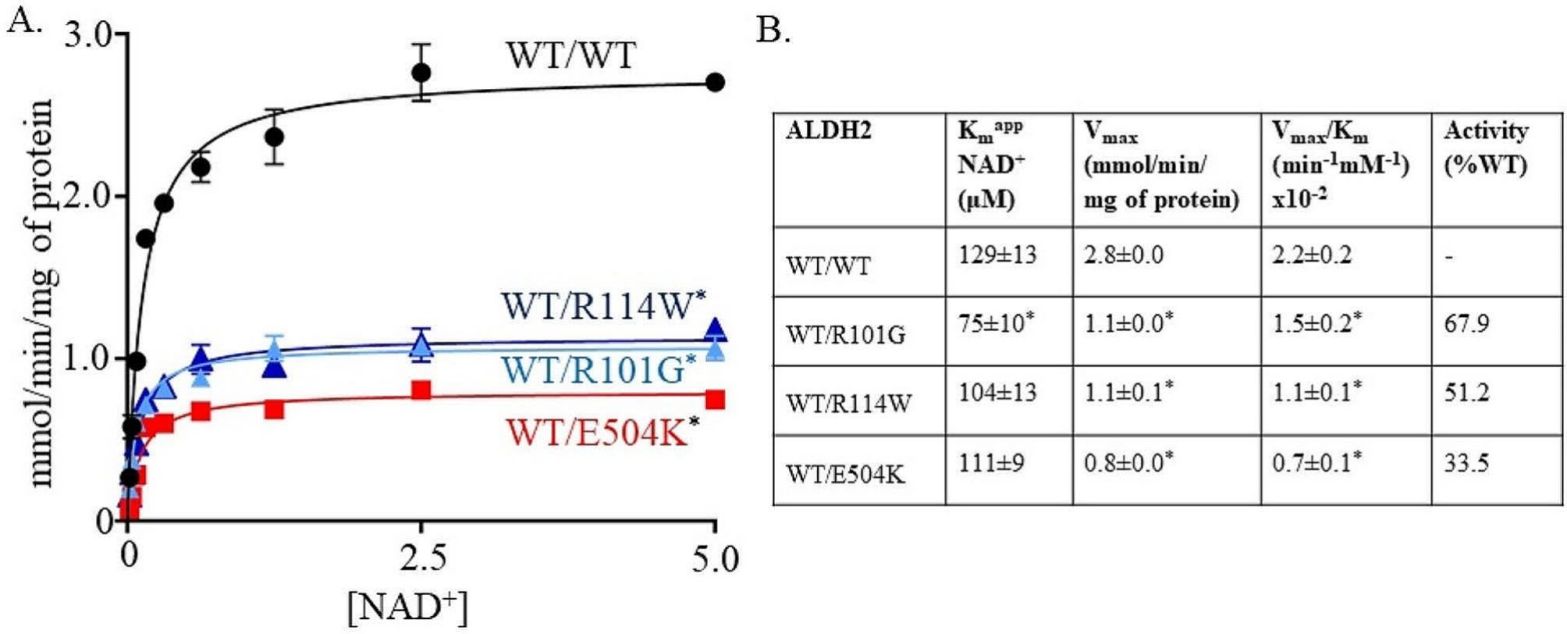
**R101G and R114W variants cause inefficient acetaldehyde metabolism. (A)** The Michaelis–Menten plot showing rate of reaction (y-axis) against NAD^+^ concentration for proteins expressed with petDuet-1 plasmid. These in vitro results support inefficient acetaldehyde metabolism for WT/R101G and WT/R114W. **(B)** V_max_ of WT/R101G and WT/R114W are significantly different from WT/E504K and wild type ALDH2. K_m_ of WT/R101G is significantly different from WT/R114W, WT/E504K and wild type ALDH2. *n* = 5/group, ^*^p < 0.01 versus wild type ALDH2 enzyme

To mimic the heterozygous condition in humans, we co-expressed in bacteria wild type and the ALDH2 variants into the pETDuet-1 vector. The co-expressed variants R101G and R114W with wild type ALDH2 had a significant decrease in V_max_ compared to wild-type ALDH2 (Fig. 5A and B, 1.1 ± 0.0^*^ and 1.1 ± 0.1^*^ versus 2.8 ± 0.0 mmol/min/mg of protein for wild type ALDH2, respectively, *n* = 5/group). In addition, E504K co-expressed with wild type ALDH2 had a significant decrease in V_max_ relative to wild type ALDH2 (Fig. 5A and B, 0.8 ± 0.0^*^ versus 2.8 ± 0.0 mmol/min/mg of protein for wild type, *n* = 5/group). Furthermore, the K_m_ for R114W co-expressed with wild-type ALDH2 was similar to wild type ALDH2 and E504K (Fig. 5B, K_m_ = 104 ± 13 versus 129 ± 13 and 111 ± 9 µM for wild type ALDH2 and E504K, respectively). However, R101G co-expressed with wild type ALDH2 had a ~ 0.3-0.4-fold decrease of K_m_ compared to wild type ALDH2 (Figs. 5B and 75 ± 10* vs. 129 ± 13 µM).

**Fig. 6:**
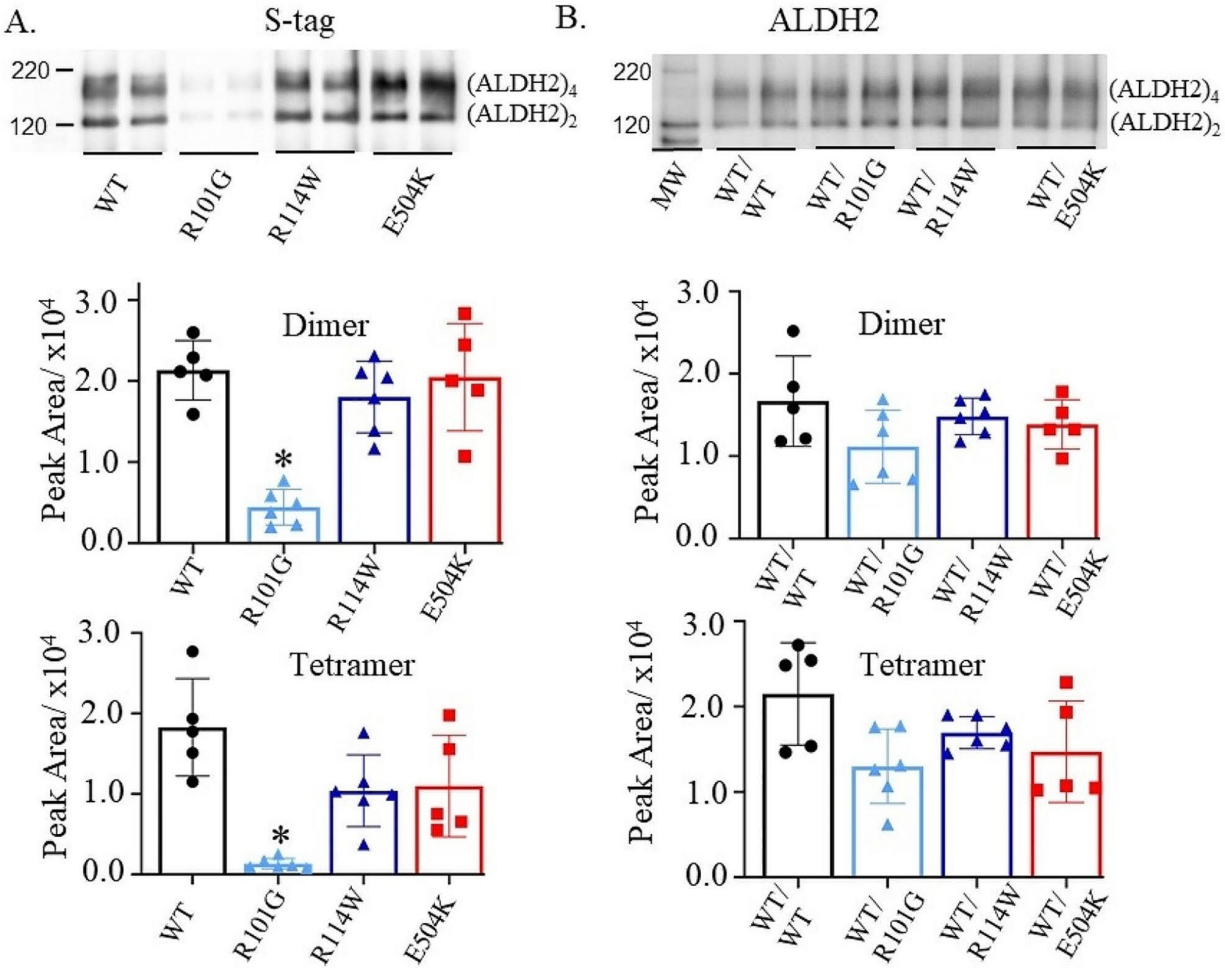
**Formation of ALDH2 dimers and tetramers. (A)** Representative western blot of wild type ALDH2 and the ALDH2 variants using a S-tag antibody with quantification of dimer and tetramer formation detected by the S-tag antibody. The R101G variant did not form dimers and tetramers. **(B)** Representative western blot of wild type ALDH2 and the ALDH2 variants using an ALDH2 antibody with quantification of dimer and tetramer formation by the ALDH2 antibody. MW = molecular weight markers, *n* = 5 or 6/group, ^*^p < 0.01 by one-way ANOVA relative to wild type, R114W and E504K

ALDH2 exists as a tetramer comprising of four identical subunits [35]. The tetramer functions as a dimer of dimers, where each of the four monomeric subunits contains a dinucleotide (NAD^+^)-binding domain, oligomerization domain and a catalytic domain with three key cysteine residues and a glutamate, which are required for the catalysis at the active site [34]. To determine whether wild type ALDH2 or the variants can form dimers and tetramers in solution, we employed a cross linker, bis(sulfosuccinimidyl)suberate, to capture these two forms of ALDH2. Furthermore, we performed western blot analysis of the crosslinked proteins to determine the presence of S-tagged proteins expressed with the petDuet-1 plasmid. A western blot with the S-tag antibody showed that R101G formed few dimers and tetramers relative to wild type and WT/E504K (Fig. 6A and Supplementary Fig. 1A). Additionally, there was no significant difference between dimers and tetramers of WT/R114W compared to wild type or WT/E504K using the S-tag antibody (Fig. 6A). When using an ALDH2 antibody to detect proteins expressed in the His-tag and S-tag cloning sites of petDuet-1 plasmid, WT/R101G formed dimers and tetramers albeit at a lower level compared wild type or WT/E504K (Fig. 6B and Supplementary Fig. 1B). However, WT/R114W had similar levels of dimers and tetramers relative to the wild type ALDH2 or the WT/E504K variant.

**Fig. 7:**
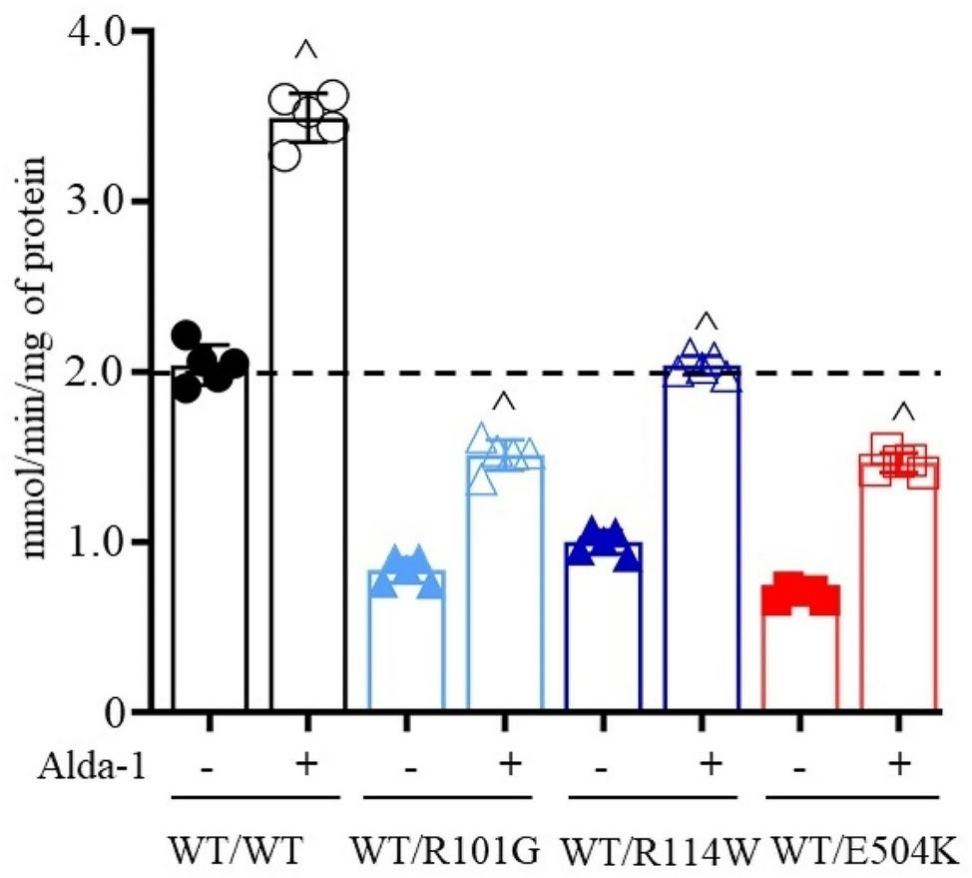
**Alda-1 enhances the activity of wild type ALDH2 and the ALDH2 variants.** The activity of wild type ALDH2 and ALDH2 variants expressed with petDuet-1 plasmid measured in the presence of Alda-1 (20µM) or vehicle. R101G and R114W activities were increased by ~ 82% and ~ 104% relative to vehicle. In addition, activity of wild type ALDH2 and E504K was increased by ~ 71% and ~ 110% relative to vehicle, *n* = 5/group, ^^^*p* < 0.01 with unpaired two-tailed Student’s t-test relative to untreated

To determine whether Alda-1, a specific ALDH2 activator, increases the activity of the variants, we measured the activity of purified enzymes in the presence of Alda-1 (20µM). Alda-1 significantly enhanced the activities of wild-type ALDH2 and E504K relative to vehicle (Fig. 7, 2.0 ± 0.1^ and 0.7 ± 0.0^ versus 3.5 ± 0.1 and 1.5 ± 0.1 mmol/min/mg of protein for wild type and E504K). In addition, Alda-1 significantly enhanced the activities of R101G and R114W relative to vehicle (Fig. 7, 0.8 ± 0.1^ and 1.0 ± 0.1^ versus 1.5 ± 0.1 and 2.0 ± 0.1 mmol/min/mg of protein for R101G and R114W, respectively). Therefore, Alda-1 brought the activity of these less-active variants close to the activity of wild type ALDH2 enzyme.

**Fig. 8:**
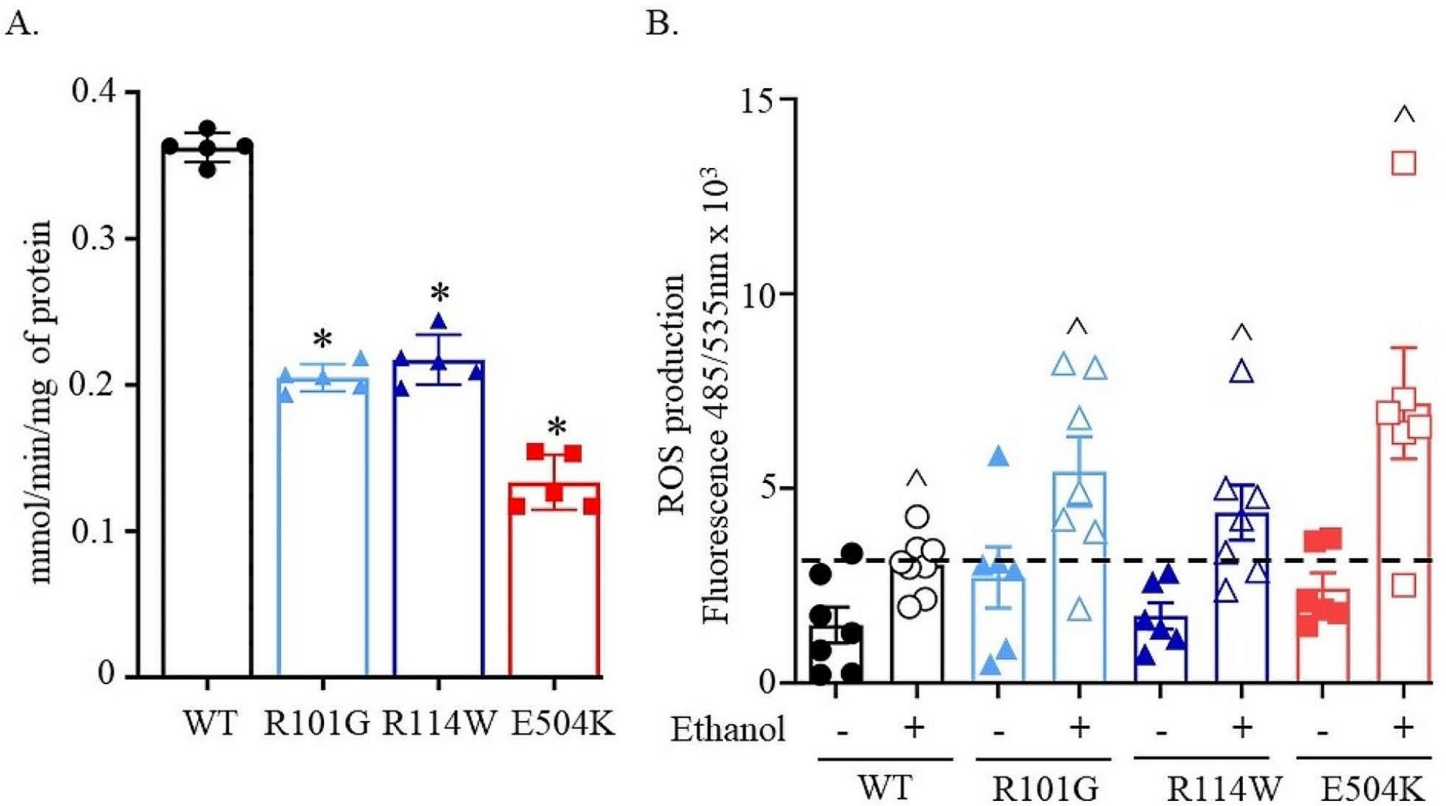
**ALDH2 variants display a reduced enzymatic activity and increased cellular ROS. (A)** ALDH2 activity in transiently transfected NIH-3T3 cells. *n* = 5/group, ^*^p < 0.01 by one-way ANOVA versus wild type ALDH2. **(B)** ROS levels measured by DFCA in cells treated 50 mM ethanol and compared with untreated cell, *n* = 6 or 7/group, ^^^*P* < 0.01 with unpaired two-tailed Student’s t-test relative to untreated

To elucidate the cellular consequences of the reduced activity ALDH2 variants, we transiently transfected NIH-3T3 cells with wild-type ALDH2 and the three ALDH2 variants. Interestingly, the activity of R101G and R114W variants transiently transfected in NIH-3T3 cells was approximately 50% lower than that of wild-type, and was slightly higher than E504K (Fig. 8A, 0.2 ± 0.0* and 0.2 ± 0.0*, respectively versus 0.4 ± 0.1 mmol/min/mg of protein for wild type).

Next, we measured cellular ROS in a NIH-3T3 cell line overexpressing wild-type ALDH2 and the three ALDH2 variants. Our results showed a marked increase in cellular ROS for the wild-type and the three variants when 3T3 cells were treated with 50 mM ethanol compared to cells treated with vehicle (Fig. 8B). Ethanol treatment led to a 2-fold increase in cellular ROS for wild-type transfected cells relative to vehicle (Figs. 8B and 3036 ± 301^ vs. 1489 ± 458, respectively, relative florescence units). All three variants demonstrated a higher than 2-fold increase in cellular ROS production upon exposure to ethanol relative to vehicle (Fig. 8B, R101G:5434 ± 913^ vs. 2712 ± 784, R114W:4377 ± 836^ vs. 1725 ± 337, and E504K:7188 ± 1426^ vs. 2431 ± 402, relative florescence units, respectively). The baseline ROS levels for R101G were also increased compared to WT transfected cells.

## Discussion

By developing a non-invasive human assay to quantify the physiological response and acetaldehyde levels after alcohol consumption, we identified ALDH2 genetic variants *rs747096195* (R101G) and *rs190764869* (R114W) cause facial flushing and a 2-fold increase in acetaldehyde following alcohol consumption. Expectantly, carriers of *rs671* (E504K) also triggered facial flushing and a 6-fold acetaldehyde accumulation compared to those without an ALDH2 variant. As acetaldehyde accumulation after alcohol consumption leads to higher risks of aerodigestive tract and esophageal cancer [12, 24, 36], this assay can more precisely provide a better understanding of alcohol-associated cancer risk in humans by quantifying genetic differences that are present in acetaldehyde metabolism.

Compared to prior research, using selective ion flow mass spectrometry (SIFT-MS) to detect breath levels of acetaldehyde after alcohol consumption have several advantages. As opposed to using gas chromatography to measure acetaldehyde [37, 38], SIFT-MS can be performed without additional sample processing, in real-time, and as a high-throughput assay. Further, SIFT-MS can detect acetaldehyde levels in the parts per billion range, providing sensitivity equivalent to laser spectroscopy [39, 40]. SIFT-MS also identified differences in acetaldehyde metabolism occurring for less common ALDH2 genetic variants, such as ALDH2 R101G and R114W. By using SIFT-MS to quantify acetaldehyde levels over a time-course after alcohol consumption, we also identified that heart rate changes correlate with breath acetaldehyde levels after an alcohol challenge. This builds upon prior human volunteer studies that describe an increased heart rate after an alcohol challenge [33, 41, 42]. Therefore, heart rate and acetaldehyde levels after alcohol consumption can provide biomarkers in humans to understand individual genetic differences in alcohol metabolism.

We identified that ALDH2 R101G and R114W variants limit acetaldehyde metabolism by two distinct means using complementary biochemical techniques with in vitro and cell culture studies. The R101G variant causes a disruption of the dimer-dimer interaction and the R114W variant causes a disruption of a H-bonding interaction within the same subunit. This is opposed to the limited enzymatic activity for the E504K variant which is attributed to the disruption of hydrogen bonding interactions at the dimer interface and coenzyme NAD^+^ binding site [34]. In the case of E504K variant, Alda-1, an allosteric ALDH2 agonist, increases acetaldehyde metabolism by providing structural stability to the enzyme, which rescues hydrogen-bond perturbations caused by the E504K variant [43]. Alda-1 binds near the exit of the substrate binding tunnel and can also significantly enhance ALDH2 activity for the R101G and R114W variants [43]. However, Alda-1 is not a universal activator for all ALDH2 variants as Alda-1 did not improve the activities of ALDH2*3 (I41V), ALDH2*6 (V304M) and ALDH2*7 (R339W) [23]. Rather than activating ALDH2 to increase acetaldehyde metabolism, targeting ALDH3A1 with a small molecule Alda-89 to enable ALDH3A1 to metabolize acetaldehyde can be an alternative approach to improve acetaldehyde metabolism for carriers of less active ALDH2 variants [44].

The less active R101G and R114W ALDH2 variants also produce higher levels of ROS when transfected cells are exposed to alcohol. This is consistent with a prior study finding the less active ALDH2 variants I41V, P92T, T244M, V304M, and R338W generate higher levels of ROS after alcohol treatment [23]. Further, even without alcohol exposure, basal levels of ROS were higher for ALDH2*2 knock-in rodents in the tongue, lung, heart, kidney, and brain tissues with respect to wild type ALDH2 mice [45, 46]. Further, heterozygote ALDH2*2 iPSC endothelial cells have higher levels of ROS production compared to their genome-edited isogenic iPSC endothelial cell lines resulting in 1460 differentially expressed genes related to ROS, angiogenesis, and inflammatory pathways [47]. Taken together, exposure to alcohol in carriers of less active ALDH2 variants can lead to higher ROS levels that modulate cellular pathways creating an oncogenic environment [48].

Besides the link between alcohol consumption and cancer, the association of alcohol use and cardiovascular disease continues to be a focus of research as elevated levels of ROS can drive cellular pathways that lead to cardiovascular disease [49]. When ALDH2 knockout mice are exposed to alcohol for 6 weeks, higher levels of ROS within the ALDH2 knockout mice with respect to wild type ALDH2 mice were associated with reduced left ventricular ejection fraction and increased myocardial fibrosis [50]. A study with diabetic-induced ALDH2 knockout mice showed that ALDH2 deficiency increases diabetes-induced oxidative stress by increasing the lipid peroxidation product, 4-hydroxynonenal [51]. However, studies suggest in humans the ALDH2*2 genetic variant limits excessive alcohol drinking, and the impact of ALDH2 genetic variants on cardiovascular disease may be mitigated [52]. For example, when examining the association of alcohol consumption with carotid plaque burden in 22,384 adults from the China Kadoorie Biobank, individuals with the wild type ALDH2 genotype had a higher association with carotid plaque burden relative to carriers of the ALDH2*2 genetic variant [53]. These findings are also supported by a study examining coronary artery calcification in 1029 Japanese men who consume alcohol where carriers of the ALDH2*2 variant had a lower coronary artery calcification burden relative to those without the ALDH2*2 variant [54]. A 12-year observational study of 512,000 adults within China Kadoorie Biobank revealed an association between alcohol consumption with 61 different diseases but genetic evidence whether the ALDH2*2 variant modified this risk was limited in scope due to inadequate statistical power [55]. Together, these studies suggest that the interplay between genetics and alcohol consumption is complex and requires future experimental and translational research to understand the interplay between alcohol, ALDH2 genetic, and cardiovascular disease.

The health risks of alcohol use may also be influenced by congeners within alcoholic beverages that are produced during ethanol production by fermentation or distillation. The types of congeners formed include phenols, esters, and ketones in addition to alcohols, such as methanol and butanol, and acetaldehyde. Unregulated beverages high in congeners consumed in East and Southern Africa such as chang’aa, gongo, and kachasu, may contribute to why Eastern and Southern Africa has the second highest risk of esophageal cancer in the world [17, 56]. In addition, home-made and fruit-based spirits have higher acetaldehyde content relative to grain-based or industry produced spirits [57]. Further, congeners can also intensify hangover symptoms [58, 59] and spirits with less congeners such as vodka produce less hangover symptoms in study participants relative to whiskey [60]. Taken together, the impact of congeners on health risks such as cancer and cardiovascular disease require further investigation.

Our results need to be interpreted within the realm of potential limitations. We focused on ALDH2 variants instead of alcohol dehydrogenase (ADH) which converts alcohol to acetaldehyde. As the activity of ALDH2 correlates with ADH activity in the liver [61], the subtle differences within groups for our study could be due to genetic variability of ADH. As our study did not consider examining mitochondrial localization of ALDH2 or the ALDH2 variants, we measured total cellular ROS instead of mitochondrial ROS. Alcohol can also cause DNA damage via ROS and acetaldehyde which was not measured for the ALDH2 variants identified in this study. As the recruitment flyers were designed to identify people that have facial flushing after they consume alcohol, it is untested whether acetaldehyde accumulation may occur without facial flushing. Furthermore, variants other than *rs671* are relatively rare in the general population, which prevented the collection of a large sample size for *rs747096195* (R101G) and *rs190764869* (R114W) that we identified. Our study focused on how ALDH2 variants impact the metabolism of acetaldehyde but did not consider how congeners in alcoholic beverages may influence aldehyde metabolism. Regardless, the non-invasive assay we developed can provide individualized assessments of acetaldehyde metabolism; leading to more personalized medicine strategies by identifying those that have impaired acetaldehyde metabolism after alcohol consumption versus those that do not.

## Conclusions

Here we developed a methodology to non-invasively quantify acetaldehyde metabolism after alcohol consumption and identified two variants in ALDH2 besides *rs671* that cause acetaldehyde accumulation after an alcohol challenge. Future work will involve leveraging this alcohol challenge assay to further phenotype human ALDH2 genetic variants in combination with supporting in vitro and cell culture studies to more precisely define the interplay between alcohol consumption, ALDH2 genetic variants, and cancer.

### Electronic supplementary material

Below is the link to the electronic supplementary material.

Supplementary Material 1

## Data Availability

All data generated or analyzed during this study are included in this published article.

## Abbreviations

ALDH2: Aldehyde Dehydrogenase 2
DCFDA: 2,7-Dichlorofluorescein Diacetate
ROS: Reactive Oxygen Species
IRB: Institutional Review Board
SIFT-MS: Selective Ion-Flow Mass Spectrometry
