# Supplementary material for: Uncovering newly identified aldehyde dehydrogenase 2 genetic variants that lead to acetaldehyde accumulation after an alcohol challenge
Source: J Transl Med. 2024 Jul 29;22:697. doi: 10.1186/s12967-024-05507-x (PMC11288122; doi:10.1186/s12967-024-05507-x)
Supplement: Supplementary file 1 — Supplementary Material 1 [file 12967_2024_5507_MOESM1_ESM.pdf]

SUPPLEMENTAL MATERIAL

for

Uncovering newly identified aldehyde dehydrogenase 2 genetic variants that lead to acetaldehyde accumulation after an alcohol challenge

Freeborn Rwere[1], Joseph R. White[1], Rafaela C.R. Hell[1], Xuan Yu[1], Xiaocong Zeng[1], Leslie McNeil[1], Kevin N. Zhou[1], Martin S. Angst[1], Che-Hong Chen[2], Daria Mochly-Rosen[2], Eric R. Gross[1*]

[1]Department of Anesthesiology, Perioperative and Pain Medicine, School of Medicine, Stanford University, Stanford, CA 94305, USA

[2]Department of Chemical and Systems Biology, School of Medicine, Stanford University, Stanford, CA 94305, USA

***Correspondence:** Eric R. Gross, Stanford University, School of Medicine, Department of Anesthesiology, Perioperative and Pain Medicine, Telephone: 650-723-7442, Fax: 650-725-5840, E-mail: ergross@stanford.edu

Supplemental Figure 1.

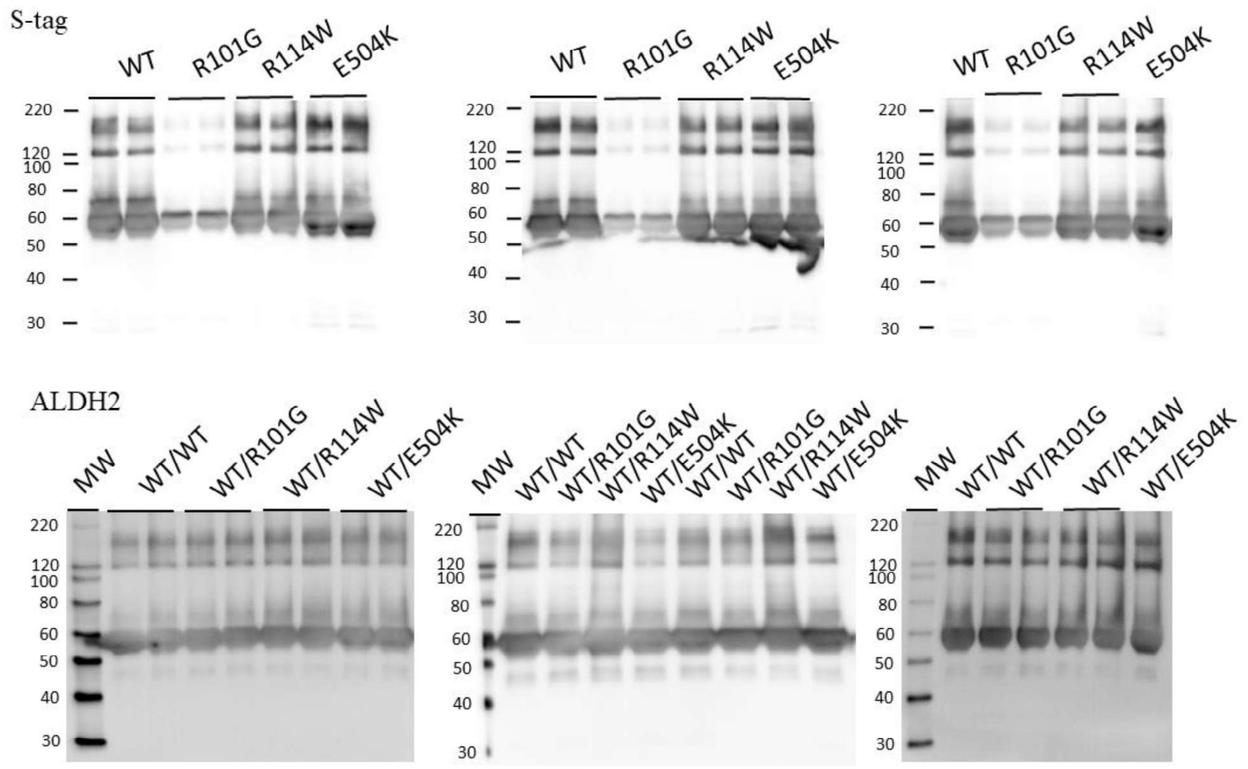

Supplemental Figure 1. Western blots for ALDH2 quantification from two different protein batches per enzyme. **A.** S-tag western blots. The R101G variant did not form ALDH2 dimers and a tetramer, whereas other ALDH2 variants expressed in the S-tag site formed dimers and tetramers. **B.** Western blots probed with an ALDH2 antibody. Both ALDH2 dimers and tetramers were detected. MW = molecular weight markers

Supplemental Table 1. ALDH2 primers for exome sequencing

Exon	Forward Primer (5'- to -3')	Reverse Primer (5'- to -3')	Amplicon size/bp	Annealing temperature/ °C
1	TAGCGCCACCCGCTTCGCTTGCATCA	TGCGGGGGACTCGGGCCGGAAAACAAA	351	58
2	GGTAGTCAGTATTAGGTTGACAGCTGG	TTAACACCGTTGTCTGCAGACAAG	311	58
3	TGTGCAGCGATATGCTGATGACC	GCTGCGAGCCTATTCACCACAT	283	58
4	TTGGAGAGACCATGGCAATAGTCCAG	TTTACCTCCTAACAACGTTGCCTCCC	324	58
5	CAGAAAGACTCAGCTGGACCAGTTTG	TTGTCAGAGCCCATCTTCTTACCTGCC	344	58
6	CCAGTGTAGTTCTCTGAGGAAGCT	TAAGAGGGAGCCACTCTGGTTCACAT	332	58
7	GACCACATGTGTCCTTGGCAGA	GGAGCTCACCTTGAGCATGTCGT	318	58
8	TCTCTCGTGGTCCAGTTGCTCACT	ACAGCAACCAACGCCATTGGGCA	328	58
9	AGCAGGCATCAACCCTTACAGT	TTCTCATGCTGGCTCTAGACA	347	58

10	CTGCATAATTCTAAG CCTGAAGCCT	CATCAGGATGCCAGGC TGAACTGTTT	318	58
11	TTCCCCTGGAACTGT TAGAGCATGGCT	ATTCCAGGATGGTGAC CACCAGATTC	376	58
12	GTCCTGGGAGTGTAA CCCATAA	AACAGACCCCAATCCC CCAGCA	266	58

Supplemental Table 2. Demographics including age, weight, and sex of participants given an alcohol challenge

	Wild type	*rs671*	*rs747096195*	*rs190764869*
Number of participants	8	8	1	1
Age (years)	28±2	29±2	60	25
Weight (pounds)	147±8	146±8	138	143
Gender (Male/Female)	4/4	4/4	0/1	0/1
Smoker	None	None	None	None

Supplemental Table 3. Soft ionization parameters for selective ion flow mass spectrometry

Metabolite	Reagent Ion	Reaction Ratio	Mass (m/z)
Acetaldehyde	H_3O^+	3.7×10^{-9}	45, 63
	NO^+	6.0×10^{-9}	43, 61

Supplemental Table 4. Sequences of oligonucleotide primers used to mutate ALDH2

**ALDH2**	**Forward Primer (5'- to -3')**	**Reverse Primer (5'- to -3')**	**Amplicon size/bp**	**Annealing temperature**
R101G	GACGCATCACACGGGGG CCGGCTGCTG	CAGCAGCCGGCCCCC GTGTGATGCGTC	27	55°C
R114W	CTGATCGAGTGG GAC CGGACCTACCTGGCG	CGCCAGGTAGGTCCA GTCCCGCTCGATCAG	30	55°C
E504K	GGGCTGCAG GCATAC ACTAAGGTGAAAACTGT C	GACAGTTTTCACCTT AGTGTATGCCTGCAG CCC	33	55°C
